# Presence of hyphae in chromoblastomycosis examinations: an enigma to be solved^☆^^☆☆^

**DOI:** 10.1016/j.abd.2020.09.008

**Published:** 2021-05-15

**Authors:** Gustavo de Sá Menezes Carvalho, Karina Baruel de Camargo Votto Calbucci, Rute Facchini Lellis, John Verrinder Veasey

**Affiliations:** a Dermatology Clinic, Santa Casa de Misericórdia, São Paulo, SP, Brazil; b Pathology Laboratory, Santa Casa de Misericórdia, São Paulo, SP, Brazil

**Keywords:** Chromoblastomycosis, Diagnosis, Mycoses

## Abstract

The detection of muriform cells in direct mycological or anatomopathological examination is considered pathognomonic for chromoblastomycosis. The morphological aspect that these fungal structures acquire were called “Borelli spiders”, when associated with hyphae. Reports of this association have been described for decades, initially related to more pathogenic agents of this mycosis. More recent studies have shown aspects related to the host's immunity that participate in this process, as well as an association with a worse disease prognosis. The present study discloses the findings of complementary examinations with the presence of “Borelli’s spiders” in a patient diagnosed with chromoblastomycosis.

## Case report

**Figure 1:**
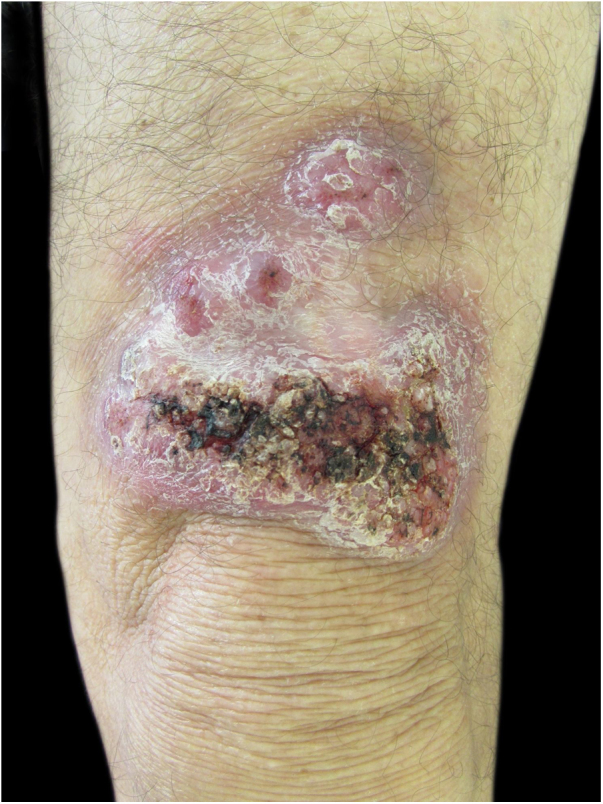
Clinical aspect on the left thigh region, characterized by an erythematous plaque, with well-defined limits, a verrucous surface and some nodular areas that measured 13 cm at the largest axis.

**Figure 2:**
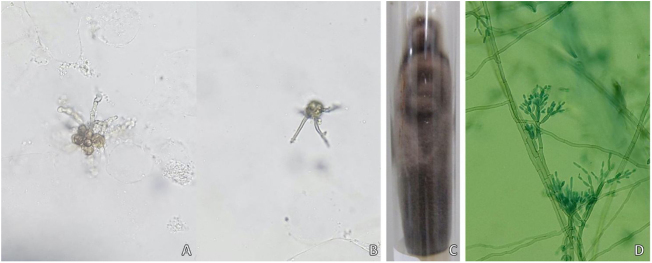
Mycological examination of a chromoblastomycosis case. (A and B), direct mycological examination (KOH 20%, ×200) showing the “Borelli spiders” in two different fields. (C and D), Fungal culture with macro-microscopic morphology compatible with *Fonsecaea pedrosoi*.

A 58-year-old male patient, born in the state of Pará, reported a lesion on the right thigh region for 15 years, characterized by an erythematous plaque, with well-defined borders, a verrucous surface and some nodular areas that measured about 13 cm in its largest axis (Fig. 1). The patient denied a history of local trauma. A direct microscopic examination was performed, which showed the presence of muriform cells or Medlar bodies (MB) (Fig. 2). Additionally, fungal culture disclosed the growth of a blackish and velvety colony, with macro-microscopic morphology compatible with *Fonsecaea pedrosoi*. The anatomopathological examination showed MB, some associated with dematiaceous hyphae, which received the name of “Borelli's spiders” (BS). These findings confirmed the diagnosis of chromoblastomycosis (CBM), and then treatment with itraconazole (200 mg/day for six months) was started, with no significant improvement of the lesions; therefore, it was necessary to combine other therapies (cryotherapy and surgical excision).

## Discussion

**Figure 3:**
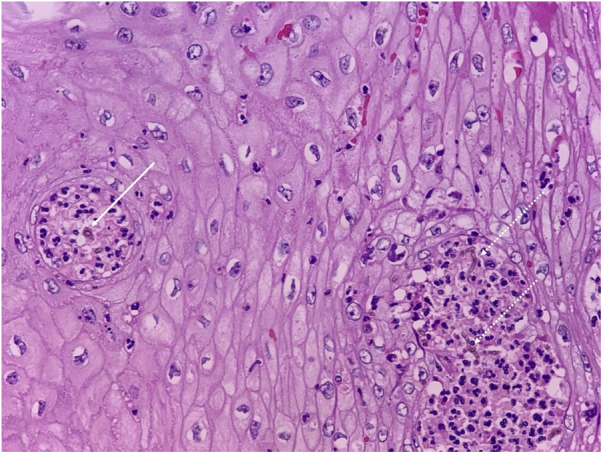
Histopathological analysis of chromoblastomycosis (Hematoxylin & eosin, ×40). Continuous arrow: muriform body (MB) in the center of an intraepidermal microabscess, pathognomonic of the disease. Dashed arrows: identification of septated dematiaceous hyphae.

CBM is a type of mycosis with chronic evolution that affects the skin and subcutaneous tissue, caused by dematiaceous filamentous fungi.^1^, ^2^ Its main causative agents are *Fonsecaea pedrosoi* and *Phialophora verrucosa*, geophilic organisms with a high prevalence in humid areas of tropical and subtropical climates.^1^, ^2^, ^3^ The most frequently affected sites are the lower limbs due to a traumatic mechanism, through the inoculation of hyphae and fungal conidia into the host’s skin.^2^, ^3^, ^4^ Once installed in the tissue, the fungus adheres to the epithelial cells and differentiates into structures called muriform, fumagoid, or sclerotic cells, also known as Medlar bodies (MB), which resist destruction by the host's effector cells, favoring the progression of the infection. The cutaneous lesions are polymorphic, including nodules, plaques, tumors, and scars.^1^, ^2^, ^3^, ^4^ The diagnosis of CBM is confirmed by the detection of MB, pathognomonic of CBM, in direct mycological and histopathological examination, regardless of the species involved (Figure 2, Figure 3).^1^, ^2^, ^3^

**Figure 4:**
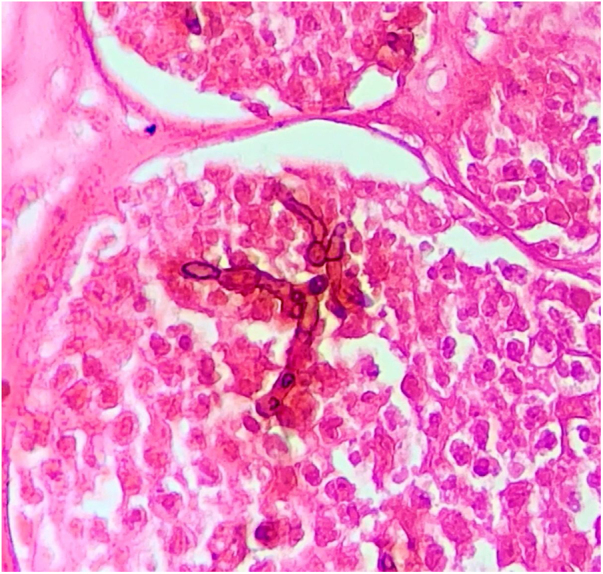
A – Histopathological analysis of chromoblastomycosis showing muriform bodies associated with dematiaceous hyphae, “Borelli spiders” (Fontana Masson, ×1000).

**Figure 5:**
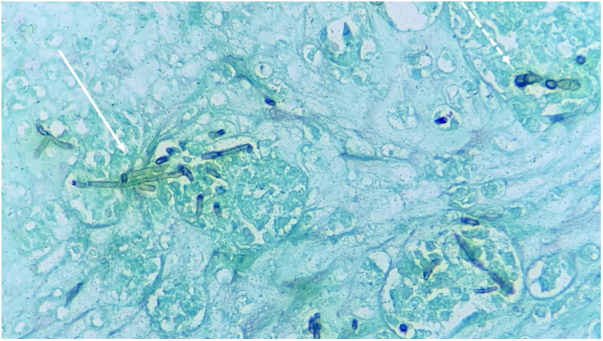
Histopathological analysis of chromoblastomycosis. Dashed arrow: muriform bodies. Continuous arrow: dematiaceous hyphae (Grocott, ×400).

Reports of dematiaceous hyphae found in association with MB have been described for decades, primarily by Borelli. The morphological aspect that the fungal structures acquire was called BS, and its finding was initially associated with the more pathogenic species of this mycosis.^5^, ^6^, ^7^, ^8^ Subsequently, this finding was associated with the species that were more resistant to therapy, being indicative of low response to classic treatments, requiring associations, dose adjustments, or surgical approaches in many cases.^1^, ^6^, ^7^ These structures are evident in both direct mycological and histopathological examinations, as seen in the present case (Figure 4, Figure 5).

In studies on CBM immunology, it was observed that the fungi are able to regulate the host's inflammatory response, preventing the inflammatory process and contributing to the adaptation and opportunism process.^9^ MBs can alter the gene expression pattern of macrophages, resulting in the histopathological characteristics observed in CBM with granulomatous inflammatory infiltrates, sometimes suppurative and with the presence of micro-abscesses. The presence of hyphae and muriform cells in the tissues increases the levels of the inflammatory cytokines TNF-α (tumor necrosis factor-alpha), IL-1β (interleukin 1-beta), and IL-6 (interleukin-6), increasing disease duration and the pathogen persistence in the tissue.^9^ This uncontrolled and chronic immune response does not provide protection for the host against the fungus and ends up leading to the maintenance of the infection.^9^ However, studies that correlate the pathophysiology related to BS and its immune response are still lacking in the literature.

The understanding of these findings becomes of the utmost importance for the treatment of CBM, which is considered a challenge, especially in the most exuberant and severe clinical forms.^2^, ^3^ The treatment of CBM is associated with low cure rates and high chances of recurrence.^1^, ^2^, ^3^ Long periods of therapy with antifungals are required, which can be associated with surgery, cryotherapy, thermotherapy, and immunotherapy.^1^, ^2^, ^10^

These data lead us to understand that the parasite/host binomial balance is affected by interference on both sides, which may favor the emergence of BS and would possibly act to make the disease chronic and hinder therapy. With the advancement of the use of immunomodulatory drugs, the possibility of finding these structures increases. Future studies on the clinical presentations and laboratory, and therapeutic expressions in these cases may help to better understand the disease and its management.

## Financial support

None declared.

## Authors’ contributions

Gustavo de Sá Menezes Carvalho: Drafting and editing of the manuscript; collection, analysis, and interpretation of data.

Karina Baruel de Camargo Votto Calbucci: Drafting and editing of the manuscript; collection, analysis, and interpretation of data.

Rute Facchini Lellis: Collection, analysis and interpretation of data; effective participation in research orientation; critical review of the literature; critical review of the manuscript.

John Verrinder Veasey: Approval of the final version of the manuscript; design and planning of the study; drafting and editing of the manuscript; collection, analysis and interpretation of data; effective participation in research orientation; critical review of the literature; critical review of the manuscript.

## Conflicts of interest

None declared.
